# Prognostic Value of Circulating MicroRNA-210 Levels in Patients with Moderate to Severe Aortic Stenosis

**DOI:** 10.1371/journal.pone.0091812

**Published:** 2014-03-13

**Authors:** Helge Røsjø, Mai Britt Dahl, Anja Bye, Johanna Andreassen, Marit Jørgensen, Ulrik Wisløff, Geir Christensen, Thor Edvardsen, Torbjørn Omland

**Affiliations:** 1 Division of Medicine, Akershus University Hospital, Lørenskog, Norway; 2 K.G. Jebsen Cardiac Research Centre and Center for Heart Failure Research, Institute of Clinical Medicine, University of Oslo, Oslo, Norway; 3 Department of Clinical Molecular Biology (EpiGen), UiO, Akershus University Hospital, Lørenskog, Norway; 4 K.G. Jebsen Centre for Exercise in Medicine, Department of Circulation and Medical Imaging, Faculty of Medicine, Norwegian Institute of Science and Technology, Trondheim, Norway; 5 Department of Cardiology, Oslo University Hospital, Rikshospitalet, Oslo, Norway; 6 Institute for Experimental Medical Research, Oslo University Hospital, Ullevål, Oslo, Norway; IRCCS-Policlinico San Donato, Italy

## Abstract

**Background:**

Circulating micro-RNAs have been proposed as a novel class of cardiovascular (CV) biomarkers, but whether they meet analytical requirements and provide additional information to establish risk indices have not been established. miR-210 levels are increased in subjects with low VO_2_ max, which is a recognized risk factor in patients with aortic stenosis (AS), and we hypothesized that circulating miR-210 levels may be increased in patients with AS and associated with a poor prognosis.

**Methods:**

We measured circulating miR-210 levels by real-time PCR in 57 patients with moderate to severe AS and in 10 age- and gender-matched healthy controls. The merit of miR-210 as a biomarker was assessed according to established criteria, including by comparing miR-210 levels with NT-proBNP and miR-22 levels, which is another miRNA biomarker candidate.

**Results:**

All patients and control subjects had miR-210 levels within the range of detection (Cq<35) and the analytical variability was low. Circulating miR-210 levels were 2.0±0.2 [mean±SEM] fold increased in AS patients compared to controls (p = 0.002), whereas miR-22 levels were not differently expressed in the AS patients (0.12±0.06 fold increase, p = 0.45). The increase in miR-210 levels in AS patients was comparable to the increment in NT-proBNP levels: [AUC] 0.82 (95% CI 0.70–0.90) vs. 0.85 (0.75–0.93), respectively, p = 0.71. During a median follow-up of 1287 days, 15 patients (26%) died. There was a significant association between higher circulating levels of miR-210 and increased mortality during follow-up: hazard ratio [supra- vs. inframedian levels] 3.3 (95% CI 1.1–10.5), p = 0.039. Adjusting for other risk indices in multivariate analysis did not attenuate the prognostic merit of circulating miR-210 levels.

**Conclusion:**

Circulating miR-210 levels are increased in patients with AS and provide independent prognostic information to established risk indices. Analytical characteristics were also excellent supporting the potential of micro-RNAs as novel CV biomarkers.

## Introduction

Current risk stratification in patients with aortic stenosis (AS) is based on symptoms (dyspnea, syncope, chest pain) and the identification of aortic valve orifice narrowing by echocardiography [1]. However, as the majority of patients with AS are elderly, a large proportion of the patients have a sedentary life style, causing a delay in the recognition of symptoms [1]. Hence, irreversible myocardial damage may occur before the patients experience symptoms, and additional means to identify high-risk patients are needed [2].

MicroRNAs (miRNAs) are short (∼22) nucleotide non-coding RNAs that influence cell function by regulating messenger-RNA stability and function [3]. miRNAs have the ability to regulate multiple downstream protein targets, and individual miRNAs have been found to be of crucial importance for myocardial function [3]. Moreover, the release of miRNAs to the circulation and the transport of miRNAs throughout the body bound to proteins [4], lipids [5], or as part of microvesicles [4] suggest that miRNAs may represent a new class of cardiovascular (CV) biomarkers [6]. With individual miRNAs regulating multiple downstream protein pathways, it has been postulated that individual miRNAs may provide integrated information on several pathophysiological axes [3]. However, although miRNAs seem to have potential as CV biomarkers, it is important that all biomarker candidates are examined according to established criteria [7]. These criteria include analytical characteristics (accuracy and reproducibility) and that the biomarker provides incremental information to the information that can be obtained from established risk indices. Currently, few studies have assessed miRNA biomarkers according to these criteria. miRNAs also cluster in networks based on common physiological properties and most previous miRNA biomarker studies have explored several miRNAs in combination [8], [9]. From a statistical point of view, the multimarker approach carries the risk of false positive results due to the testing of several biomarkers in the same cohort. Moreover, a multimarker approach is less user-friendly as several biomarkers need to be assayed and considered together. Hence, we believe that for biomarker research the exploration of individual miRNAs is important. An interesting miRNA biomarker candidate is miR-210 as miR-210 production is closely linked to cellular hypoxia [10]. We recently also found circulating miR-210 levels to be the most strongly increased miRNA in subjects with low VO_2_ max of the 720 miRNAs that were tested [11]. The close association between circulating miR-210 levels and aerobic fitness suggest that miR-210 may have merit as a prognostic biomarker in patients with cardiovascular disease (CVD) and especially in patients with aortic stenosis (AS) as aerobic capacity is an important risk factor and used for selection to surgery in AS [12]. Accordingly, in this study we wanted to assess whether circulating miR-210 levels fulfill the proposed criteria for novel biomarkers in a cohort of patients with moderate to severe AS.

## Results

### Clinical and Echocardiographical Data of AS Patients and Control Subjects

The patients and control subjects were evenly matched on age, gender, and body mass index (BMI) (Table 1). Only a minority of the AS patients were considered to be in New York Heart Association (NYHA) functional class III/IV, but N-terminal pro-B-type natriuretic peptide (NT-proBNP) levels were significantly increased in AS patients compared to control subjects. The proportion of comorbidity was high in the AS patients, and this was also reflected in medication use among the AS patients.

**Table 1:** Baseline characteristics of the patients with aortic stenosis and the control subjects.

	AS (n = 57)	Ctr (n = 10)	p
**Age (y)**	75±1	74±1	0.72
**Women**	31 (54%)	3 (30%)	0.19
**BMI**	26±1	25±1	0.47
**NYHA functional class III/IV**	19 (33%)		
**Creatinine clearance (mL/min)**	70 (52–86)		
**Coronary artery disease**	38 (67%)		
**Hypertension**	29 (51%)		
**Diabetes mellitus**	8 (14%)		
**Atrial fibrillation**	12 (21%)		
**COPD**	5 (9%)		
**Medication**			
** β-blocker**	27 (47%)		
** Ca^2+^ channel blocker**	9 (16%)		
** ACEI**	10 (18%)		
** AII-blocker**	11 (19%)		
** Aspirin**	32 (56%)		
** Warfarin**	11 (19%)		
** Diuretics**	25 (44%)		
** Statin**	34 (60%)		
** Aldosterone antagonist**	6 (11%)		
**NT-proBNP (pg/mL)**	785 (334–2395)	156 (56–342)	<0.001
**miR-210 levels (fold change vs. ctr)**	3.0±0.2	1.0±0.5	0.002
**miR-22 levels** **(fold change vs. ctr)**	1.12±0.06	1.00±0.18	0.45

BMI, body mass index; NYHA class, New York Heart Association functional class; COPD, chronic obstructive pulmonary disease; ACEI, angiotensin-converting enzyme inhibitor; AII-blocker, angiotensin II blocker; NT-proBNP, N-terminal pro-B-type natriuretic peptide; and miR, micro-RNA.

Compared to the control subjects, patients with AS had reduced aortic valve orifice area and increased velocity and pressure gradient over the valve, evidence of left ventricular (LV) hypertrophy, and impaired LV relaxation as assessed by E/e’ (Table 2).

**Table 2:** Echocardiographical data in the patients with aortic stenosis and control subjects.

	AS (n = 57)*	Ctr (n = 10)	p
**Left atrial area (cm^2^)**	22.4±0.9	18.4±1.6	0.07
**LV end-diastolic dimension (cm)**	5.2±0.09	5.2±0.3	0.89
**LVEF (%)**	57±1	60±1	0.45
**Fractional shortening (%)**	37.7±1.4	37.3±1.5	0.92
**LV cardiac index (L/minm^2^)**	2.7±0.07	2.7±0.2	0.79
**Aortic valve area (cm^2^)**	0.7±0.03	3.2±0.2	<0.001
**Aortic valve velocity_max_ (m/s)**	4.5±0.2	1.3±0.07	<0.001
**Aortic valve mean pressure gradient (mmHg)**	56±4	4±0.4	<0.001
**Interventricular septal end-diastolic dimension (cm)**	1.2±0.03	0.9±0.06	<0.001
**LV end-diastolic posterior wall dimension (cm)**	1.0±0.02	0.8±0.04	0.002
**E/e’**	19.1±1.7	8.0±0.8	0.009
**E/A ratio**	1.0±0.1	0.8±0.06	0.32
**Mitral valve deceleration time (ms)**	236±10	239±19	0.90
**Relative wall thickness**	0.43±0.01	0.33±0.01	<0.001
**LV mass (g)**	232±10	169±21	0.012

LV, left ventricular; and LVEF, left ventricular ejection fraction.

*n = 53 for E/e’ and n = 52 for E/A ratio.

### miR-210 Levels in Patients with AS

**Figure 1:**
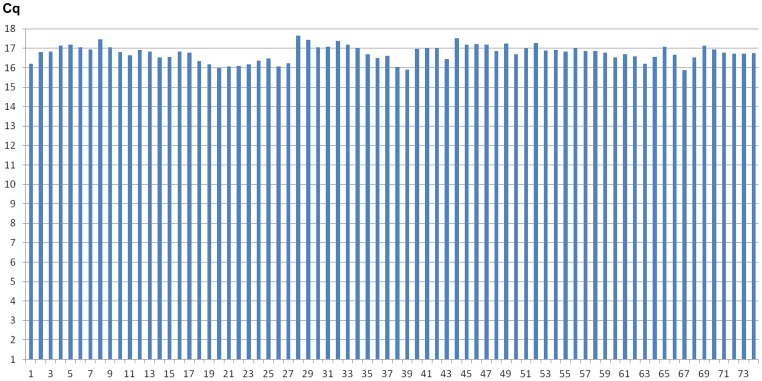
Extraction from serum and RT-qPCR of the spike-in control cel-miR-39 were performed with minimal variation between samples (CV 2.4% for AS patients [#1–36 and #38–58] and 2.2% for the control subjects [#59–74]), which indicates that miRNAs seem to meet the analytical requirements proposed for novel biomarkers. Data are presented as the absolute Cq values.

All patients and control subjects had miR-210 levels within the range of detection (Cq<35; range Cq 29–32). The coefficient of variation for the samples run in triplet was 0.6% for both AS patients and the control subjects. Coefficient of variations for the spike-in control synthetic C. elegans miR-39 (cel-miR-39) was 2.4% for AS patients (n = 57) and 2.2% for the control subjects (n = 10) and the raw Cq values are presented in Figure 1.

**Figure 2:**
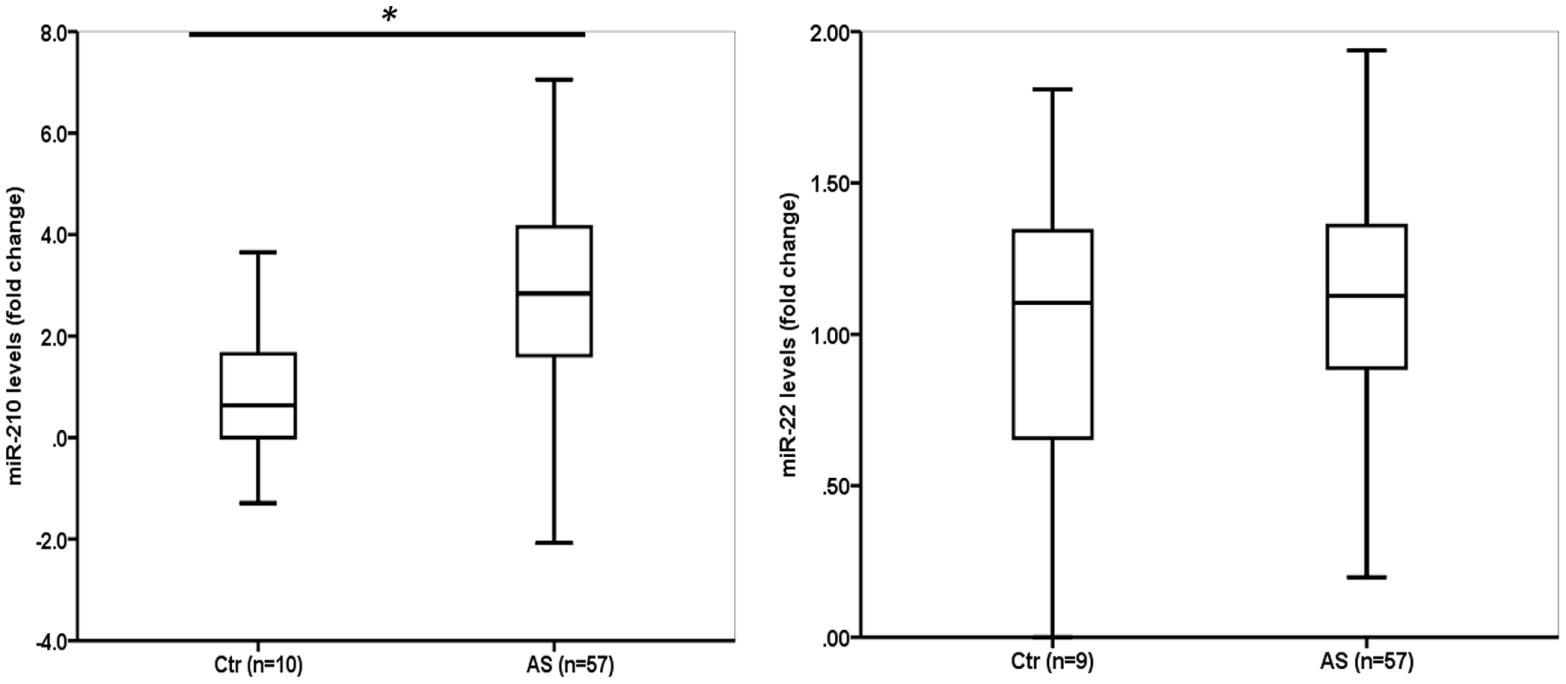
miR-210 levels were significantly increased in patients with AS compared to age- and gender-matched control subjects, whereas miR-22 levels were not altered. The horizontal line within the box represents the median level, the boundaries of the box quartiles 1–3, and the whiskers range (maximum value restricted to 1.5×interquartile range from the median). *p<0.01.

miR-210 levels were higher in AS patients compared to the control subjects: 3.0±0.2 vs. 1.0±0.5, p = 0.002 (Fig. 2). In contrast, miR-22 levels (Cq 27–30), which is another miRNA biomarker candidate, were not increased in the AS patients (Table 1, Fig. 2). The area under the curve as assessed by receiver operating characteristics (ROC-AUC) to separate AS patients from the control subjects of miR-210 levels were comparable to that of NT-proBNP levels: 0.82 (95% confidence interval [CI] 0.70–0.90) vs. 0.85 (0.75–0.93), respectively, p = 0.71. miR-210 levels were significantly correlated with miR-22 levels in the AS patients (r = 0.46, p<0.001), but not in the control subjects (Table 3). There was also a modest but significant inverse correlation between miR-210 levels and LV end-diastolic dimension in patients with AS. No other significant correlations were found between clinical and echocardiographical variables in AS patients or control subjects. As assessed by logistic regression, there were no significant associations between miR-210 levels over the median and clinical variables, including presence of coronary artery disease (CAD), nor echocardiographical variables, although the associations with left atrial area (odds ratio [OR] 0.92 [95% CI 0.84–1.003], p = 0.06) and NT-proBNP levels (OR 1.54 [0.98–2.41], p = 0.06) were of borderline significance. miR-210 levels in patients with and without CAD were 3.1±0.4 vs. 2.8±0.3 (fold change vs. control subjects), respectively, p = 0.56.

**Table 3:** Correlations in AS patients and control subjects (Ctr) between miR-210 levels and clinical and echocardiographical parameters of myocardial structure and function.

Variable	AS patients (n = 57)*		Ctr (n = 10)	
	r	p	r	p
**Age**	0.22	0.10	0.18	0.62
**Gender**	−0.06	0.64	−0.42	0.23
**BMI**	−0.13	0.33	−0.05	0.89
**Creatinine Clearance**	−0.15	0.26	–	–
**NT-proBNP levels**	0.18	0.18	−0.34	0.33
**miR-22 levels**	0.46	<0.001	−0.14	0.72
**Left atrial area (cm^2^)**	−0.23	0.08	−0.55	0.10
**LV end-diastolic dimension (cm)**	−0.26	0.049	−0.45	0.20
**LVEF (%)**	0.009	0.95	−0.21	0.57
**Fractional shortening (%)**	−0.08	0.55	0.36	0.31
**LV cardiac index (L/minm^2^)**	−0.11	0.41	−0.54	0.31
**Aortic valve area (cm^2)^**	−0.09	0.49	−0.20	0.58
**Aortic valve velocity_max_ (m/s)**	−0.05	0.74	−0.44	0.20
**Aortic valve mean pressure gradient (mmHg)**	−0.008	0.95	−0.05	0.89
**Interventricular septal end-diastolic dimension (cm)**	0.006	0.97	−0.04	0.92
**LV end-diastolic posterior wall dimension (cm)**	−0.03	0.85	−0.23	0.52
**E/e’**	0.02	0.86	0.14	0.71
**E/A ratio**	0.16	0.27	0.04	0.91
**Mitral valve deceleration time (ms)**	−0.19	0.16	0.30	0.39
**Relative wall thickness**	0.16	0.25	0.40	0.25
**LV mass (g)**	−0.14	0.29	−0.33	0.35

BMI, body mass index; NT-proBNP, N-terminal pro-B-type natriuretic peptide; and miR, micro-RNA; LV, left ventricular; and LVEF, left ventricular ejection fraction.

*n = 53 for E/e’ and n = 52 for E/A ratio.

### Prognostic Utility of miR-210 Levels in Patients with AS

**Figure 3:**
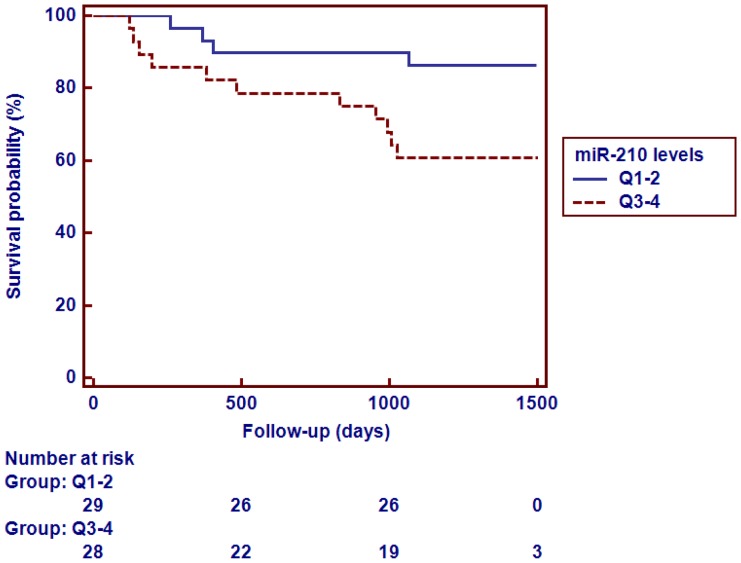
Assosciation between miR-210 levels and mortality during follow-up with miR-210 levels divided by the median (p = 0.029 by the log-rank test).

During a median follow-up of 1287 days (quartile [Q] 1–3 1045–1400 days), 15 (26%) of the AS patients died. A miR-210 level over the median was associated with increased risk of mortality (p = 0.029 by the log-rank test, Fig. 3). High NT-proBNP and miR-210 levels, together with history of hypertension and increasing eccentric LV hypertrophy based on calculation of relative wall thickness, were associated with mortality in multivariate Cox proportional hazard regression analysis (Table 4). The prognostic accuracy of miR-210 for all-cause mortality was comparable to the accuracy of NT-proBNP levels: AUC = 0.64 (95% CI 0.50–0.76) vs. AUC = 0.67 (0.53–0.79), respectively, p = 0.83.

**Table 4:** Predictors for mortality during follow-up by univariate and multivariate Cox proportional hazard regression analysis.

*Univariate analysis*			
	Hazard ratio	95% CI	p
**Age, per 1 y increase**	1.06	0.99–1.14	0.09
**Male gender**	2.60	0.89–7.63	0.08
**BMI, per 1 unit increase**	0.91	0.80–1.04	0.16
**NYHA functional class III/IV**	1.13	0.39–3.32	0.82
**Creatinine clereance, mL/min**	0.98	0.96–1.003	0.08
**Coronary artery disease**	1.52	0.49–4.79	0.47
**Hypertension**	0.29	0.09–0.90	0.032
**Diabetes mellitus**	0.93	0.21–4.11	0.92
**Atrial fibrilliation**	0.22	0.03–1.70	0.15
**COPD**	3.91	1.09–14.1	0.037
**Medication**			
** β**–**blocker**	0.95	0.34–2.62	0.92
** Ca^2+^ channel blocker**	0.32	0.04–2.45	0.27
** ACEI**	1.86	0.59–5.85	0.29
** AII**–**blocker**	0.034	0.001–6.20	0.20
** Aspirin**	1.65	0.56–4.83	0.36
** Warfarin**	0.25	0.03–1.91	0.18
** Diuretics**	1.12	0.41–3.19	0.83
** Statin**	0.96	0.34–2.69	0.94
** Aldosterone antagonist**	2.57	0.72–9.16	0.15
**Left atrial area (cm^2^)**	0.98	0.91–1.07	0.09
**LV end**–**diastolic dimension (cm)**	1.85	0.87–3.93	0.11
**LVEF (%)**	0.96	0.92–1.009	0.11
**Fractional shortening (%)**	0.96	0.92–1.008	0.10
**LV cardiac index (L/minm^2^)**	0.88	0.32–2.48	0.81
**Aortic valve area (cm^2^)**	6.77	0.81–56.78	0.08
**Aortic valve velocity_max_ (m/s)**	0.74	0.47–1.17	0.20
**Aortic valve mean pressure gradient (mmHg)**	0.99	0.97–1.009	0.26
**Interventricular septal end-diastolic dimension (cm)**	0.37	0.02–5.69	0.47
**LV end-diastolic posterior wall dimension (cm)**	0.037	0.002–0.88	0.041
**E/e’**	1.03	1.00–1.07	0.08
**E/A ratio**	0.55	0.16–1.91	0.35
**Mitral valve deceleration time (ms)**	1.00	0.99–1.007	0.93
**Relative wall thickness** **(per 0.1 increase)**	0.44	0.21–0.92	0.028
**LV mass (g)**	1.00	0.99–1.007	0.92
**Aortic valvular surgery**	0.10	0.03–0.34	<0.001
**NT-proBNP levels**	1.71	1.04–2.81	0.036
**miR-210 levels (supramedian)**	3.34	1.06–10.49	0.039
***Multivariate analysis***			
**Hypertension**	0.21	0.07–0.70	0.011
**Relative wall thickness** **(per 0.1 increase)**	0.39	0.18–0.85	0.018
**NT-proBNP levels**	2.20	1.17–4.14	0.014
**miR-210 levels (supramedian)**	4.79	1.40–16.45	0.013

BMI, body mass index; NYHA class, New York Heart Association functional class; COPD, chronic obstructive pulmonary disease; ACEI, angiotensin-converting enzyme inhibitor; AII-blocker, angiotensin II blocker; LV, left ventricular; LVEF, left ventricular ejection fraction NT-proBNP, N-terminal pro-B-type natriuretic peptide; and miR, micro-RNA.

NT-proBNP levels were transformed by the natural logarithm prior to regression analysis due to a right-skewed distribution.

## Discussion

The principal results of this study are (1) only minor variation for the spike-in control cel-miR-39 across different samples, (2) circulating miR-210 levels are increased in patients with AS, and (3) circulating miR-210 levels were associated with the risk of mortality during follow-up, independently of established risk indices. miRNA biomarkers thus seem to meet the requirements for analytical accuracy and reproducibility, and our data support circulating miR-210 as a novel CV biomarker that should be further tested in larger patient groups.

To the best of our knowledge, our study is the first to examine miRNA biomarkers in a stringent manner according to the criteria proposed by Morrow and de Lemos [7]. Analytical accuracy and reproducibility are key factors when determining whether a biomarker may have clinical potential. We found the analytical stability to be excellent when measuring cel-miR-39 levels across different samples of AS patients and in the control subjects. The coefficient of variations of 2.4% and 2.2%, respectively, are well within the range that we also see for commercially available protein assays, including previously reported coefficient of variations for two high-sensitivity troponin assays in our laboratory [13]. Furthermore, cDNA synthesis and quantitative real-time PCR (RT-qPCR) analyses demonstrated minimal variability with a mean coefficient of variation of 0.6% for the samples assayed in triplet. Interestingly, we also found cel-miR-39 expression to be in the same range as previous reported for Taqman-based analyses [14]. Pertinent to this, although miRNA raw Cq levels are known to be highly variable according to RNA extraction and miRNA detection techniques, miR-210 and miR-22 levels in our study were well within the range of detection with Cqs of 29–32 and 27–30, respectively. We used relative quantification, and miR-210 levels are presented as fold change to the levels in the control subjects, which is the strategy also employed by other groups [8]. We found circulating miR-22 levels to be unaltered in patients with AS compared to the control subjects, which makes it unlikely that the increment in miR-210 levels is a result of a general upregulation of all circulating miRNAs in AS. Analogous to our previous publication on circulating miRNAs and physical capacity [11], we normalized miR-210 levels against miR-425 levels. We chose miR-425 levels as the normalization strategy as we previously have found miR-425 to be expressed closest to the mean Cq of all miRNAs (geomean) in two screening studies exploring 720 miRNAs (34 subjects in total) with correlation coefficients of r = 0.93 (n = 24) and r = 0.94 (n = 10) between geomean and miR-425 Cq values, respectively (Figure S1). Hence, in our experience miR-425 is the miRNA that best reflects the total miRNA expression in an individual. Leading researchers in the field also advocate a combination of a spike-in control to assess extraction quality and the use of a stable miRNA for normalization [15], [16]. Furthermore, no study has yet reported circulating miR-425 levels to be specifically influenced by CVD, and other groups have also proposed miR-425 as a normalization strategy [17].

We found miR-210 levels to provide incremental prognostic information to established risk indices in patients with AS, which is a *sine qua non* criterion for a biomarker to have clinical potential [7]. Thus, miR-210 levels seem to provide information on pathophysiology not covered by the established CV risk indices. The additional information obtained by measuring miR-210 levels is also demonstrated by the lack of associations between miR-210 levels and clinical and echocardiographical variables and NT-proBNP levels in linear regression analysis. Currently, no information is available concerning the cellular source responsible for the increased circulating miR-210 levels in AS. However, both the endothelium and the myocardium may contribute to circulating miR-210 levels as these tissues increase their miR-210 production during cellular hypoxia and injury [10], [18]. In contrast, platelets do not seem to be an important contributor to miR-210 levels [19]. Of note, miR-425 does not appear to be a platelet-derived miRNA, which supports our use of miR-425 as a normalization strategy for miRNA biomarkers.

Other groups have also found circulating miR-210 levels linked to CVD as miR-210 levels are increased in proportion to the severity of heart failure [20], [21]. Circulating miR-210 levels have also been found elevated in patients with myocardial infarction [22] and peripheral artery disease [23], and to provide prognostic information independently of established risk indices in patients with acute kidney injury [24]. In heart failure, a model for circulating miR-210 levels as reflective of the mismatch between impaired cardiac function and the oxygen demand of peripheral organs has been postulated [21]. Our recent report of increased circulating miR-210 levels in subjects with low aerobic fitness lends some support to the model of hypoxia-mediated miR-210 increment. Hence, local supply-demand imbalance in the myocardium due to LV hypertrophy could be a factor leading to higher miR-210 levels in patients with AS [25]. This model needs to be further explored in additional clinical and experimental studies, but is supported by previous experimental investigations demonstrating hypoxia inducible factor-1α regulated miR-210 expression during cellular hypoxia [26]. Accordingly, miR-210 is most likely not a specific biomarker for AS, but rather a CV risk marker upregulated by and associated with prognosis in patients with AS. Additional studies are needed to confirm this model for miR-210, but this would be analogous to most other CV biomarkers, including NT-proBNP that are increased by several CV etiologies and provide prognostic information across the spectrum of CVD [27]. Whether miR-210 levels may improve patient management, which is the final criteria for novel biomarkers [7], cannot be assessed based on the available data in the literature or this study. miRNA biomarkers are still in the early stage of testing and additional and larger studies are clearly needed to address this question.

This study has both strengths and limitations. We performed biomarker assessment according to established criteria [7]. The low coefficient of variations of the miRNA triplet and the spike-in cel-miR-39 control validate the small-RNA extraction protocol employed and the robust technical work regarding RNA extraction and real-time PCR in our study. The extensive echocardiographical examination and the benchmarking against NT-proBNP levels for prognostic assessment also represent strengths of our work. In contrast, the modest number of patients represents a limitation. However, from a statistical point of view the substantial event rate (26% mortality) during follow-up is considered of more importance than the absolute number of patients included in the study [28]. Furthermore, our final multivariate Cox regression model includes, in addition to high miR-210 levels, established risk factors in AS such as NT-proBNP levels and relative wall thickness, thus supporting the validity of our work. We acknowledge that there is no generally accepted normalization strategy for miRNA biomarker studies. A common alternative strategy has been to normalize against the spike-in control cel-miR-39, but we would argue against this strategy. As demonstrated in this study, the spike-in control presents with a very stable Cq value, whereas the geomean may differ by 2–3 Cqs from individual to individual (Figure S1). Accordingly, when normalizing against cel-miR-39 levels subjects with a low overall miRNA expression (and thus a high geomean Cq value) will be found to have low or normal levels of the specific miRNA of interest, although in reality this specific miRNA could be increased compared to the expression of other miRNAs of the subject. Cel-miR-39 has also been demonstrated to differ according to medication [14], which reduces the quality of cel-miR-39 as the normalization strategy.

In conclusion, we report that miR-210 may have potential as a novel CV biomarker as analytical variation for RNA extraction and RT-qPCR analysis was low and circulating miR-210 levels provided prognostic information independently of established risk indices, including NT-proBNP levels. Accordingly, additional studies of circulating miR-210 levels in larger patient cohorts are needed to further explore miR-210 as a CV biomarker.

## Methods

### Patient Cohort and Control Subjects

The study was approved by the South-Eastern Norway Regional Ethics Committee and Oslo University Hospital, Rikshospitalet and performed according to the Declaration of Helsinki. All patients provided written informed consent prior to study commencement.

We recruited 57 patients with AS referred to a European tertiary cardiothoracic surgery centre for evaluation. The patient recruitment was finalized in 2009 prior to the start of transcatheter aortic valve implantation therapy, thus only open heart surgery was available as a therapeutic option in our patients. We recruited the patients during pre-operative assessment and thus prior to the final decision concerning surgery. Patients with acute coronary syndromes or primarily aortic regurgitation or right-sided valvular heart disease were not included. Information regarding medical history, current symptoms, medication, and functional class was obtained from the medical records, as previously reported [29]. CAD was defined as either previous acute myocardial infarction, previous percutaneous coronary intervention, previous coronary artery bypass grafting, or a stenosis ≥50% of an epicardial artery on angiography. Data regarding mortality until August 1, 2012 were obtained from electronic hospital records, which are synchronized with Statistics Norway on a monthly basis.

We also recruited 13 age- and gender-matched control subjects for this study. The control subjects were recruited outside of the hospital, had no previous history of CVD or diabetes mellitus, and no current symptoms of CVD, as evaluated by one researcher (HR). Material for RNA extraction was available from 10 of the control subjects.

### Echocardiographical Examination

The patients were examined by a standard protocol for transthoracic echocardiography using a Vivid 7 (GE Healthcare, Horten, Norway). All images were digitally stored for later offline analysis (Echopac, GE Vingmed). We obtained standard parasternal long axis and three apical views recordings in the end-expiratory phase with the subjects in supine left lateral position. LV dimension, septal and posterior wall thickness, and mass were measured as recommended [30]. We assessed LV systolic function by calculating the ejection fraction (LVEF) according to the modified Simpson’s rule from biplane 4-chamber and long-axis view and by determining fractional shortening. The severity of aortic valvular orifice narrowing was assessed by measuring aortic valve velocity and calculating the mean pressure gradient and aortic valve area. LV diastolic function was assessed by pulsed Doppler transmitral peak early (E), peak late (A) and E deceleration time. We recorded early diastolic velocity (e’) at the base of the septal and lateral mitral annulus. All the recordings were reviewed by one researcher (TE) with no knowledge of circulating biomarker levels.

### Blood Sampling and Protein Biochemical Analyses

Blood sampling in serum and EDTA tubes was performed by standard venepuncture at the time of echocardiography for the patients and control subjects. Blood samples were immediately put on ice, processed <30 min, and stored at −80°C prior to transport to Akershus University Hospital for further analyses. We measured NT-proBNP by the proBNP II assay (Roche Diagnostics, Penzberg, Germany) in EDTA samples and we calculated creatinine clearance by the Cockcroft-Gault formula.

### miR Isolation

RNA was extracted from serum according to the protocol recommended by Exiqon (Vedbæk, Denmark). In brief, we first made QIAzol Master Mix by adding 1.25 µL 0.8 µg/µL MS2 bacterophage RNA to 800 µL QIAzol (QIAGEN Sciences, Germantown, MN). We then added 750 µL QIAzol Master Mix to 200 µL serum and extracted miRs by the use of chloroform, ethanol, spin columns, and RNase free water. Cel-miR-39 was included as a spike-in control to assess the efficiency of RNA extraction.

### Real-time PCR Analysis

We synthesized complementary DNA (cDNA) from 4 µL RNA in a total reaction volume of 20 µL using the miRCURY LNA™ Universal RT microRNA PCR, Polyadenylation and cDNA synthesis kit (Exiqon). miR-210, -22, -425, and cel-miR-39 levels were measured by RT-qPCR employing pre-made (cat#204333, cat#204606, and cat#204337) and custom-made primer assays from Exiqon and using the HT 7900 Real-Time PCR System (Applied Biosystems, Foster City, CA). We normalized miR-210 levels to miR-425 levels as miR-425 recently was reported as a circulating miRNA for normalization [17] and we have found miR-425 to be expressed closest to the geomean in two screening studies exploring 720 miRNAs in total (n = 34) (Figure S1 demonstrating r = 0.93 and r = 0.94 for correlations between miR-425 and geomean levels in the two screening cohorts). miR-425 was also our normalization strategy in a previous miRNA biomarker study [11]. For each serum sample, three cDNA synthesis reactions were performed, and all cDNA samples were included for RT-qPCR analysis. Samples were run on 96 well plates and inter-plate calibration for the assays were based upon the use of two individuals included in all plates. Relative quantification calculations were performed according to the 2–[delta][delta]Ct method [31] by the use of SDS 2.4 software (Applied Biosystems). The relative microRNA expression is shown as fold change values (log2 of RQ values), which is the strategy to report miRNA levels also used by other groups [8].

### Statistical Analysis

Continuous data are presented as mean (± SEM) except for NT-proBNP levels that are presented as median (Q 1–3) due to a right-skewed distribution (assessed by the Kolmogorov-Smirnov one sample test). We examined between group differences by the Student’s *t* test or the Mann-Whitney *U* test as appropriate. Categorical data are presented as absolute numbers and percentages, and were compared by the Chi-square or the Fisher Exact test. Correlations were assessed by the Pearson method. Variables associated with a high miR-210 levels were examined by logistic regression analysis with age, gender, BMI, NYHA functional class (I/II vs. III/IV), comorbidities, echocardiographical variables, estimated creatinine clearance, and NT-proBNP levels (logarithmical transformed) included in the model. Kaplan-Meier plots with patients subdivided according to median miR-210 levels were generated and crude risk compared by the log-rank test. Adjusted risk estimates of miR-210 levels above the median and established clinical and echocardiographical risk factors were assessed by Cox proportional hazard regression analysis. Diagnostic and prognostic accuracy were assessed by receiver operating characteristics (ROC) curve analysis [32] with area under the curve (AUC) presented with 95% CI. P-values <0.05 were considered significant for all analyses. Statistical analyses were performed with SPSS for Windows version 19.0 (SPSS, Chicago, IL).

## Supporting Information

Figure S1 **We normalized against miR-425 levels as we previously have found this endogenous miRNA expressed closest to the mean of all circulating miRNAs (geomean) in two different screening cohorts with 720 miRs measured in total (r = 0.93 for n = 24 and r = 0.94 for n = 10, p<0.001 in both).** Accordingly, our data support miR-425 levels as a surrogate marker of the geomean and therefore miR-425 should be appropriate for normalization.
(PDF)
