# Supplementary material for: Prognostic Value of Circulating MicroRNA-210 Levels in Patients with Moderate to Severe Aortic Stenosis
Source: PLoS One. 2014 Mar 13;9(3):e91812. doi: 10.1371/journal.pone.0091812 (PMC3953554; doi:10.1371/journal.pone.0091812)

SUPPLEMENTARY MATERIAL

to

Prognostic value of circulating microRNA-210 levels in patients with moderate to severe aortic stenosis

Helge Røsjø MD, PhD[1,2]; Mai Britt Dahl MSc[1,2,3]; Anja Bye PhD[4]; Johanna Andreassen BSc[5]; Marit Jørgensen BSc[1,2,3]; Ulrik Wisløff PhD[4]; Geir Christensen MD, PhD, MHA[2,6]; Thor Edvardsen MD, PhD, MHA[2,5]; Torbjørn Omland MD, PhD, MPH[1,2]

[1] Division of Medicine, Akershus University Hospital, Lørenskog, Norway

[2] K.G. Jebsen Cardiac Research Centre and Center for Heart Failure Research, Institute of Clinical Medicine, University of Oslo, Oslo, Norway

[3] Department of Clinical Molecular Biology (EpiGen), UiO, Akershus University Hospital, Lørenskog, Norway

[4] K.G Jebsen Centre for Exercise in Medicine, Department of Circulation and Medical Imaging, Faculty of Medicine, Norwegian Institute of Science and Technology, Trondheim, Norway

[5] Department of Cardiology, Oslo University Hospital, Rikshospitalet, Oslo, Norway

[6] Institute for Experimental Medical Research, Oslo University Hospital, Ullevål, Oslo, Norway

Supplementary Figure S1.

Cohort #1 (n=24)

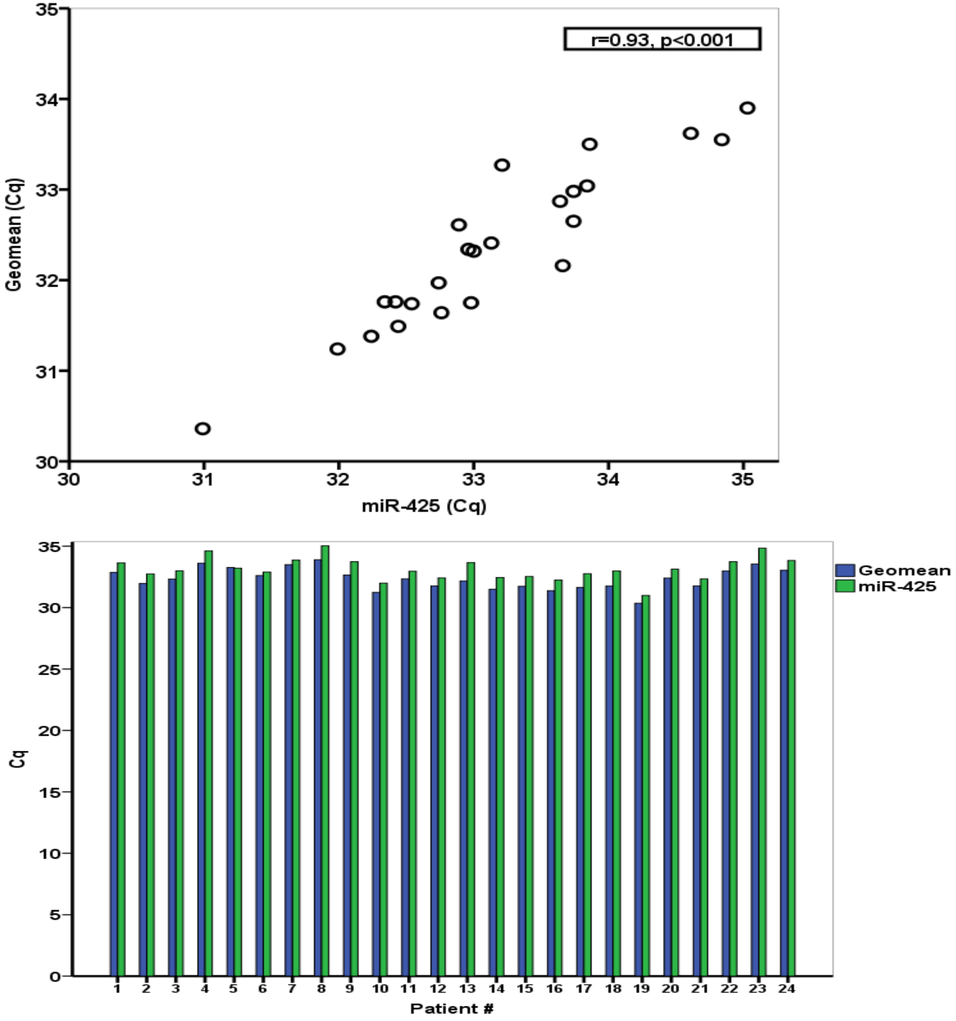

Cohort #2 (n=10)

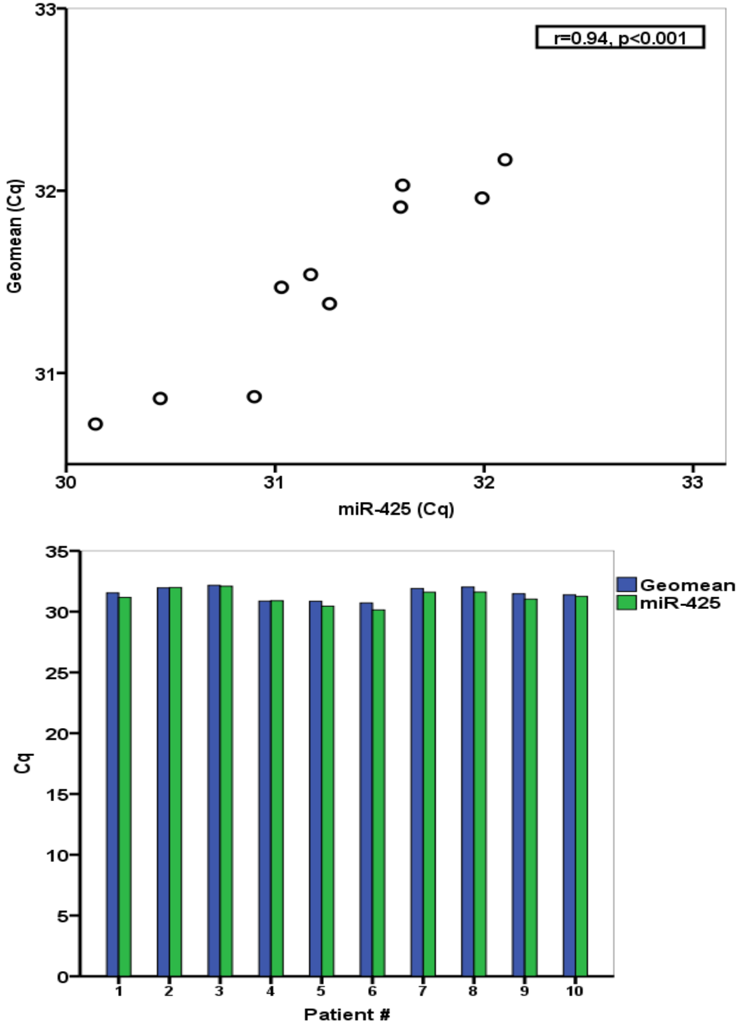

Supplement: Figure S1 — We normalized against miR-425 levels as we previously have found this endogenous miRNA expressed closest to the mean of all circulating miRNAs (geomean) in two different screening cohorts with 720 miRs measured in total (r = 0.93 for n = 24 and r = 0.94 for n = 10, p<0.001 in both). Accordingly, our data support miR-425 levels as a surrogate marker of the geomean and therefore miR-425 should be appropriate for normalization. (PDF) [file pone.0091812.s001.pdf]
